# Gp120 on HIV-1 Virions Lacks O-Linked Carbohydrate

**DOI:** 10.1371/journal.pone.0124784

**Published:** 2015-04-27

**Authors:** Elizabeth Stansell, Maria Panico, Kevin Canis, Poh-Choo Pang, Laura Bouché, Daniel Binet, Michael-John O'Connor, Elena Chertova, Julian Bess, Jeffrey D. Lifson, Stuart M. Haslam, Howard R. Morris, Ronald C. Desrosiers, Anne Dell

**Affiliations:** 1 New England Primate Research Center, Harvard Medical School, Southborough, Massachusetts, 01772, United States of America; 2 Department of Life Sciences, Imperial College London, South Kensington Campus, London, SW7 2AZ, United Kingdom; 3 MS-RTC (Mass Spectrometry Research and Training Centre), Suite 3.1 Lido Medical Centre, St. Saviours Road, Jersey, JE2 7LA, United Kingdom; 4 AIDS and Cancer Virus Program, Leidos Biomedical Research, Inc., Frederick National Laboratory, Frederick, Maryland, 21702, United States of America; Institute of Infection and Global Health, UNITED KINGDOM

## Abstract

As HIV-1-encoded envelope protein traverses the secretory pathway, it may be modified with N- and O-linked carbohydrate. When the gp120s of HIV-1 NL4-3, HIV-1 YU2, HIV-1 Bal, HIV-1 JRFL, and HIV-1 JRCSF were expressed as secreted proteins, the threonine at consensus position 499 was found to be O-glycosylated. For SIVmac239, the corresponding threonine was also glycosylated when gp120 was recombinantly expressed. Similarly-positioned, highly-conserved threonines in the influenza A virus H1N1 HA1 and H5N1 HA1 envelope proteins were also found to carry O-glycans when expressed as secreted proteins. In all cases, the threonines were modified predominantly with disialylated core 1 glycans, together with related core 1 and core 2 structures. Secreted HIV-1 gp140 was modified to a lesser extent with mainly monosialylated core 1 O-glycans, suggesting that the ectodomain of the gp41 transmembrane component may limit the accessibility of Thr499 to glycosyltransferases. In striking contrast to these findings, gp120 on purified virions of HIV-1 Bal and SIV CP-MAC lacked any detectable O-glycosylation of the C-terminal threonine. Our results indicate the absence of O-linked carbohydrates on Thr499 as it exists on the surface of virions and suggest caution in the interpretation of analyses of post-translational modifications that utilize recombinant forms of envelope protein.

## Introduction

The envelope proteins of human immunodeficiency virus type 1 (HIV-1), simian immunodeficiency virus (SIV), and influenza A virus are synthesized as a precursor protein that must be cleaved by a cellular protease to function in viral entry [1–3]. Furin cleavage of the HIV-1-encoded envelope precursor protein gp160 results in the gp120 surface subunit and the gp41 transmembrane subunit [4]. The gp120 surface component engages receptors and the gp41 transmembrane component is responsible for fusion following receptor engagement. The primary furin cleavage site of gp160 is C-terminal to arginine at position 511 of the HIV-1 HXB2 strain (HIV-1 HXB2 R511) in the consensus numbering system [5]. While two amino acids are subsequently removed from the C-terminus of the gp120 by a carboxypeptidase [6], we will refer to the amino acid corresponding to HIV-1 HXB2 R511 as the C-terminus of gp120 [5]. Approximately ten percent of the HIV-1 gp160 precursor protein is cleaved at a secondary subtilisin-like dibasic cleavage site C-terminal to HIV-1 HXB2 R504 [7].

Many of the viral-encoded envelope proteins that traverse the secretory pathway of the cell are modified with N-linked carbohydrate and/or O-linked carbohydrate. N-linked carbohydrate is attached to the nascent protein at the asparagine of the consensus sequence N-X-S or N-X-T, where X is any amino acid except proline [8,9]. In contrast to N-linked glycosylation, there are no clear-cut consensus sequences that distinguish an O-glycosylated serine (Ser) or threonine (Thr) from a non-glycosylated Ser or Thr in the primary protein sequence. O-linked glycosylation can often be found in a Ser/Thr-rich stretch of amino acids, but an isolated Thr or Ser can also be the target of carbohydrate attachment [10]. The vastly predominant type of O-linked carbohydrate, the mucin type, begins with the attachment of N-acetylgalactosamine (GalNAc) to the target Ser or Thr [11].

Early reports suggested that the gp120 glycoprotein of HIV-1 was modified with mucin-type O-linked carbohydrate [12–14]. Enzymatic deglycosylation of HIV-1 gp120 with O-linked glycosidase resulted in increased protein mobility on SDS gels, consistent with the presence of O-linked carbohydrate on the glycoprotein [12,14]. Gel filtration analysis of carbohydrate released by β-elimination was also consistent with the presence of di/trisaccharide and monosaccharide O-linked carbohydrate on HIV-1 gp120 [13]. In preliminary mass spectrometric (MS) glycoproteomic studies, we identified a single site of attachment of disialylated core 1 and related smaller structures on Thr 499 in recombinant HIV-1 NL4-3 gp120 [15]. Our findings have recently been confirmed by reports of multiple glycosylated structures on the Thr 499 of recombinant HIV-1 gp120 [16–18].

Lectin protein from jackfruit seeds (jacalin) binds the gp120s of SIV from rhesus macaque (SIVmac), SIV from sooty mangabey (SIVsm) and some strains of HIV type 2 (HIV-2) [19]. Jacalin binds GalNAc-Ser/Thr, core 1 carbohydrate, and monosialylated core 1 [20,21]. Jacalin did not bind gp120 of mutant strain SIV04, a variant of SIVmac239 with substitutions of Ser and Thr in the V1 domain [19]. These findings were consistent with MS glycomic analyses in this same publication [19] and with earlier reports of O-linked carbohydrate in the V1 domain of gp120 for SIVmneC18 from pigtail macaques [22].

Here we describe the O-glycosylation of a highly conserved Thr near the C-terminus of secreted, recombinant, envelope, receptor-binding, surface subunit of HIV-1 (gp120), SIVmac (gp120), and influenza A virus (HA1). However, gp120 from purified virions of HIV-1 and SIVmac was totally devoid of this C-terminal O-glycosylation.

## Materials and Methods

### Plasmids and cells

TZM-bl cells were obtained from the NIH AIDS Research and Reference Reagent Program and were propagated as recommended. The source and the propagation of HEK293T cells has been previously described [19]. The parental infectious HIV-1 pNL4-3 clone was obtained from the NIH AIDS Research and Reference Reagent Program. The parental infectious SIVmac239 construct has been previously described [23].

### Construction of variant sequences

PCR methods were used to change Thr codons to serine (TCC), alanine (GCC) or valine (GTC). Fragments of DNA amplified by PCR were sequenced in full to verify the absence of off-site changes.

### Recombinant protein

For the production of gp120 protein ultilized in glycomic and glycoproteomic analyses, HEK293T cells were transfected with codon optimized histidine tagged HIV-1 NL4-3 gp120 or expression optimized histidine tagged SIVmac239 gp120 cassettes. Sixteen hours after transfection the media was exchanged with serum-free media (DMEM with 50 mM HEPES). For HIV-1 His-tagged gp120 protein, cell culture supernatant was collected 48 hours after transfection. For SIVmac239 His-tagged gp120 protein, cell culture supernatant was collected 72 hours after transfection. Cell culture supernatant was filtered through a 0.45 um polyethersulfone membrane (Nalgene). Cell-free culture supernatant containing His-tagged gp120 proteins was incubated with HIS-Select nickel affinity gel (SIGMA) and proteins were purified according to the manufacturer’s recommendation. Following elution, proteins were concentrated over a 100 kDa polyethersulfone membrane at low g force (200 × g). Proteins were then dialyzed against cold PBS pH 7.4 overnight at 4°C, separated on a 10% SDS-PAGE gel and Coomassie blue stained to determine that gp120 was the predominant protein in the preparation. As a control, HEK293T cells were mock transfected and processed in parallel. Glycomic analysis of two GNA-purified gp120s without the HIS tag yielded similar patterns as their His-tagged counterparts. The following proteins were purchased from Immune Technology: HIV-1 YU2 gp120, HIV-1 JRCSF gp120, HIV-1 Bal gp120, influenza A virus (H1N1 Puerto Rico: A/PR/8/34) HA1, and influenza A virus (H5N1 Thai: A/Thailand/2(SP33)/2004) HA1.

HIV-1 NL4-3 gp120+gp140 and HIV-1 YU2 gp120+gp140 proteins were produced from HEK293T cells transfected with codon optimized histidine tagged HIV-1 NL4-3 gp140 or HIV-1 YU2 gp140 expression vectors and purified with GNA agarose as previously described [19].

### RP-HPLC purification of virion gp120 proteins

HIV-1 and SIV virions were produced from chronically infected cell lines. Viruses are designated according to the viral strain and cell line in which they were propagated (i.e. Virus strain/Cell line, Cell log number; the AIDS and Cancer Virus Program, ACVP, Frederick National Laboratory for Cancer Research, Frederick, MD): HIV-1 BAL/Sup-T1-CCR5 CL30 CL#204 and SIV CP-MAC/Sup-T1 CL# 131. For half of the experiments infectious HIV-1 BAL/Sup-T1-CCR5 from 2.5L of cell culture supernatant was used. For the other half of the experiments HIV-1 BAL/Sup-T1-CCR5 from 2.5L of cell culture supernatant was inactivated with 2,2’-dithiodipyridine (aldrithiol-2, AT-2) prior to use [24,25]. Purified, pelleted virion samples were disrupted in 8M Guanidine-HCl (Pierce, Rockford, IL) without dithiothreitol (Calbiochem, La Jolla, CA) and fractionated by HPLC to isolate virion-derived gp120 protein. HPLC was performed at a flow rate of 300 μL/min on 2.1 x 100 mm Poros R2/H narrow bore column (Boehringer Mannheim GmbH, Germany), using aqueous acetonitrile/trifluoroacetic/acid solvents and a Shimadzu HPLC system equipped with LC-10AD pumps, SCL-10A system controller, CTO-10AC oven, FRC-10A fraction collector and SPD-M10AV diodearray detector. The gradient of buffer B (0.1% trifluoracetic acid in acetonitrile) was: 10%–36.5%, 12 min; 36.5%–37%, 4 min; 37%-41%, 7 min; 41%-70%, 12 min; and 70%, 5 min. A temperature of 55°C was maintained during HPLC separation. Peaks were detected by UV absorption at 206 and 280. Fractions containing recombinant JRCSF gp120 and SIV CP-MAC/Sup-T1 gp120 were frozen. Fractions containing HIV-1 BAL/Sup-T1-R5 gp120 were lyophilized for further SDS-PAGE purification. For SDS-PAGE purification of viral gp120 protein, gp120 purified from the HIV-1 BAL/SupT1-R5 viral samples by HPLC and the viral samples disrupted in 2x sample buffer were loaded in adjacent gel lanes on a 1.5-mm thick 4–20% Tris glycine gel (Invitrogen, Carlsbad, CA). The samples were separated by SDS-PAGE, followed by staining with Coomassie R-250. After destaining the bands containing gp120 were excised and transferred in tubes with 1% acetic acid solution.

### Mass spectrometric analyses

Samples of gp120 and hemagglutinin were reduced, carboxymethylated and digested with trypsin as described [26,27]. For glycomic experiments, the O-glycans were released by reductive elimination and permethylated prior to MS analysis as described [19]. For glycoproteomic experiments either the tryptic peptide/glycopeptide digest mixture was analysed directly by on-line nanoLC ES-MS and MS/MS or, in some experiments, the N-glycans were first removed by N-glycosidase F (Roche Applied Science) and separated from the peptides/O-glycopeptides using a Sep-Pak C18 cartridge. The latter fraction was subjected to on-line LC-MS/MS analysis as described [26–29]. PAGE gel bands of viral proteins were destained, digested with trypsin and extracted using standard protocols. Cyanogen Bromide digestion was carried out in 5% trifluoroacetic acid at room temperature overnight in the dark. Nano-LCMS and MS/MS of tryptic or CNBr digest gel extracts, and supplementary nanospray experiments on selected glycopeptides were carried out on Q* and Q-TOF quadrupole orthogonal acceleration time of flight instruments [26,27,29].

## Results

### O-glycosylation status of the highly conserved Thr for recombinant secreted HIV-1 gp120 and SIVmac239 gp120

The NetOGlyc 3.1 prediction algorithm (http://www.cbs.dtu.dk/services/NetOGlyc/) was developed utilizing known sites of mucin type O-linked carbohydrate [10]. In calculating if a serine or threonine is a potential site for the attachment of O-glycans, the amino acid content of a 31 amino acid peptide is considered together with averaged surface accessibility and the substitution matrix profile. The capability to precisely predict the substrate specificity of any one of the twenty N-acetylgalactosyl transferase proteins remains limited such that NetOGlyc3.1 correctly predicts sites of attachment for O-linked carbohydrate for approximately 70 percent of sites. A second caveat to prediction is that the occupancy of O-glycosylation can vary in vivo depending on the cells expressing the protein.

**Fig 1:**
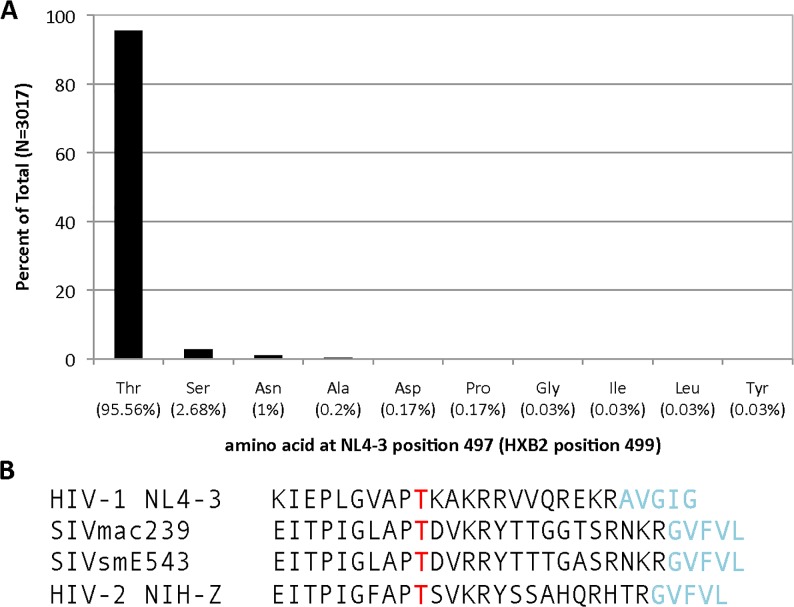
Conservation of Thr for HIV-1 and HIV-2/SIVsm/SIVmac. (A) Sequences were obtained from the HIV-1/SIVcpz alignment from the Los Alamos HIV database [5]. Sequences of Env from whole genomes of HIV-1 group M and HIV-1 group O that had a defined amino acid at HXB2 position 499 were analyzed. Shown is the rounded percentage of each amino acid: Thr (Thr), serine (Ser), asparagine (Asn), alanine (Ala), aspartic acid (Asp), proline (Pro), glycine (Gly), isoleucine (Ile), leucine (Leu), and tyrosine (Tyr). For the 3017 sequences, 2883 had Thr, 81 had Ser, 33 had Asn, 6 had Ala, 5 had Asp, 5 had Pro, and one sequence had Gly, Ile, Leu or Tyr. (B) Alignment of the C-terminus of gp120 and the N-terminus of gp41 for select viral species from the Lentivirus genus of the Retroviridae. Highly conserved Thr is denoted in red. The last R residue in black is taken as the C-terminus of gp120 by conventional usage. Light blue lettered amino acids are the N-terminus of gp41.

Analyses of gp120 sequences for five commonly used HIV-1 strains with the NetOGlyc3.1 prediction software identified a single isolated Thr as a predicted site of attachment for O-linked carbohydrate. This Thr corresponds to HIV-1 HXB2 Thr499 near the C-terminus of gp120 in the consensus numbering system [5]. To determine the extent to which this Thr is conserved, we analyzed the amino acid at the position corresponding to Thr499 of HIV-1 reference strain HXB2 from the premade alignment of HIV-1/SIVcpz in the Los Alamos HIV database [5]. The premade alignment was made from whole genome sequences available in 2011. HIV-1 sequences of group M and group O with a defined amino acid at the position corresponding to HIV-1 HXB2 Thr499 in the premade alignment were analyzed. For these 3017 Env sequences, 2883 had Thr, 81 had serine (Ser), 33 had asparagine (Asn), 6 had alanine (Ala), 5 had aspartic acid (Asp), 5 had proline (Pro), 1 had glycine (Gly), 1 had isoleucine (Ile), 1 had leucine (Leu), and 1 had tyrosine (Tyr) (Fig 1A).

For the HIV-1 group M sequences that contained this highly conserved Thr, the Thr was 13 amino acids from the C-terminus of gp120 for the vast majority of sequences (2819 of 2829), identical to the location for HIV-1 NL4-3 (Fig 1B). For the remaining Env sequences of HIV-1 group M with a corresponding Thr, the Thr is 12–18 amino acids from the C-terminus of gp120. For HIV-1 group M sequences with a serine at the position corresponding to HIV-1 HXB2 Thr499, the serine was 13 amino acids from the C-terminus of gp120 in all 81 cases. A similarly positioned Thr was found for 54 of 54 sequences of HIV-1 group O.

A corresponding Thr is found in 100% of the 72 sequences of Env from the 2011 Los Alamos HIV database premade alignment for the whole genome of HIV-2/SIVsm (Fig 1B) [5]. For SIVsm this Thr is 15–16 amino acids from the C-terminus of gp120, for SIVmac it is16 amino acids from the C-terminus of gp120, and for HIV-2 the corresponding Thr is 14–15 amino acids from the C-terminus of gp120 (Fig 1B).

**Fig 2:**
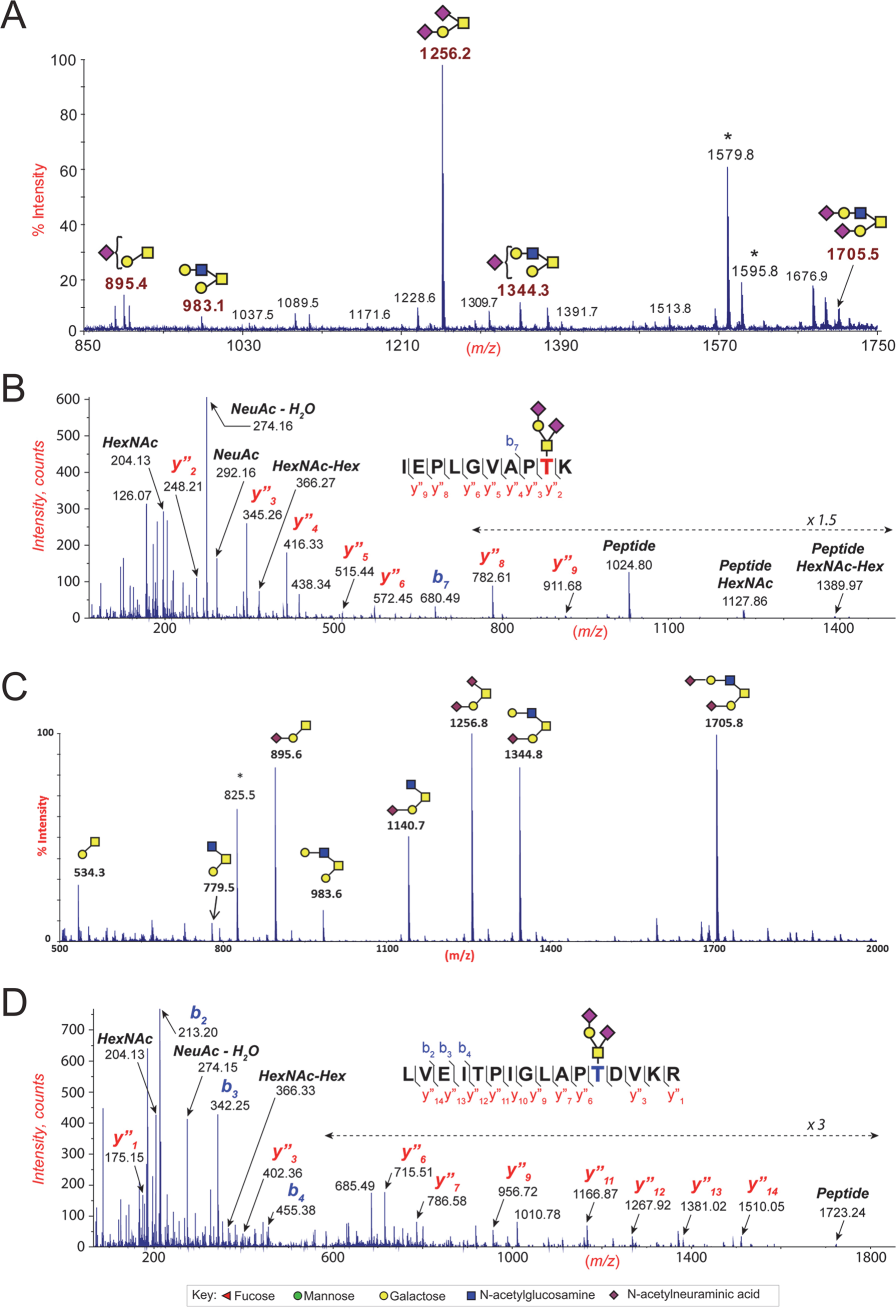
O-linked carbohydrate analyses of recombinant gp120 and gp120 purified from virions. (A) Matrix-assisted laser desorption/ionization-time-of-flight (MALDI-TOF) mass spectra of permethylated O-glycans isolated from HIV-1 NL4-3 gp120. All molecular ions are [M+Na]^+^. The sugar symbols are those employed by the Consortium for Functional Glycomics for the representation of glycan structures. Structural assignments are based on monosaccharide composition (obtained by MALDI-TOF MS), fragmentation analyses (MALDI-TOF/TOF MS/MS), and knowledge of glycan biosynthetic pathways. Asterisk denotes signals from contaminating N-glycans. (B) Electrospray Nano-LC-MS/MS spectra showing the location and attachment of the major species of O-glycan attached to Thr at position 497 in the truncated secreted HIV-1 NL4-3 gp120 (Thr499 in HXB2). (C) Matrix-assisted laser desorption/ionization-time-of-flight (MALDI-TOF) mass spectra of permethylated O-glycans isolated from the truncated secreted SIVmac239 gp120 (D) Electrospray Nano-LC-MS/MS spectra showing the location and attachment of the major species of O-glycan attached to Thr at position 510 in the truncated secreted SIVmac239 gp120.

**Fig 3:**
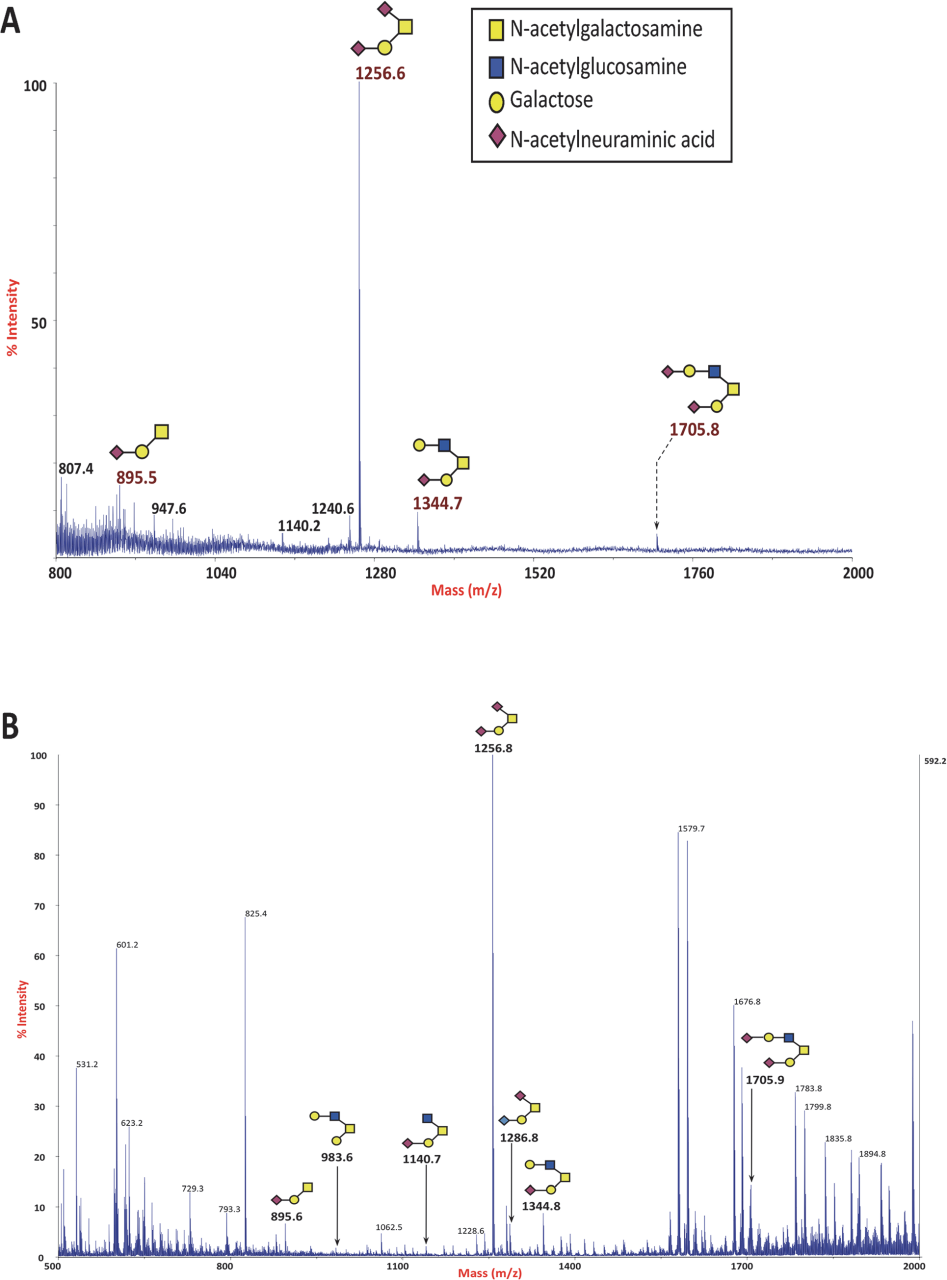
O-linked carbohydrate of recombinant gp120s from other strains of HIV-1. Matrix-assisted laser desorption/ionization-time-of-flight (MALDI-TOF) mass spectra of permethylated O-glycans isolated from gp120 of (A) HIV-1 JRCSF (B) HIV-1 YU2. See legend to Fig 1 for additional information. Signals that are not annotated are from impurities.

To determine if the highly conserved Thr at consensus position 499 of HIV-1 gp120 is modified with O-linked carbohydrate, recombinant HIV-1 gp120 was expressed in secreted form by truncation at the primary furin cleavage site and purified from the cell-free culture supernatant. Both commercial and in-house purified sources of recombinant gp120 were used. Glycomic analyses were used to analyze the total O-linked carbohydrate content for the population of purified gp120 molecules. Glycoproteomic analyses were used to identify peptides carrying the O-linked carbohydrate to determine the site of glycosylation and to estimate content of different glycosylated forms. Secreted recombinant HIV-1 NL4-3 gp120 was found in MS glycomic experiments to be modified primarily with disialylated core 1 mucin-type O-linked carbohydrate and to a lesser extent other core forms of mucin-type O-glycan structures (Fig 2A). The O-glycan on HIV-1 NL4-3 was found in glycoproteomic MS and MS/MS experiments to be covalently attached to the predicted Thr that is 13 amino acids from the C-terminus of recombinant gp120 (Fig 2B). Analyses of variant HIV-1 NL4-3 recombinant gp120s in which this Thr was substituted with alanine or valine demonstrated the absence of any other sites for O-linked carbohydrate attachment on the secreted gp120 and confirmed the sequences of the mutants. Glycomic and glycoproteomic data for secreted recombinant gp120 of HIV-1 JRCSF, HIV-1 YU2, HIV-1 JRFL, and HIV-1 BaL were similar to that for HIV-1 NL4-3, with disialylated core 1 carbohydrate attached to the C-terminal Thr being the major form (Fig 3A and 3B, Table 1, and other data not shown). For the related SIVmac239 recombinant gp120, O-linked carbohydrate primarily of the disialylated core 1 type was attached to an equivalently located Thr that is 16 amino acids from the predicted C-terminus (Fig 2C and 2D).

**Table 1:** Summary of O-glycosylation of secreted gp120s and gp120+gp140s.

HIV-1 Sample	m/z	Charge	Calculated MW	Peptide	Carbohydrate	Approximate Intensity MS
YU2 gp120	512.9	2	1023.8	IEPLGVAPTK	Native peptide	530
	695.6	2	1389.2	IEPLGVAPTK	HexNAc.Hex	500
	841.1	2	1680.2	IEPLGVAPTK	HexNAc.Hex.NeuAc	200
	986.7	2	1971.4	IEPLGVAPTK	HexNAc.Hex.NeuAc_2_	2200
	1023.7	2	2045.4	IEPLGVAPTK	HexNAc_2_.Hex_2_.NeuAc	480
	612.6	2	1223.2	IEPLGVAPTKAK	Native peptide	Not Observed
	940.2	2	1878.4	IEPLGVAPTKAK	HexNAc.Hex.NeuAc	Not Observed
	724.2	3	2169.6	IEPLGVAPTKAK	HexNAc.Hex.NeuAc_2_	Not Observed
YU2 gp120+gp140	512.7	2	1023.4	IEPLGVAPTK	Native peptide	4940
	695.2	2	1388.4	IEPLGVAPTK	HexNAc.Hex	2770
	840.7	2	1679.4	IEPLGVAPTK	HexNAc.Hex.NeuAc	500
	612.6	2	1223.2	IEPLGVAPTKAK	Native peptide	80
	940.2	2	1878.4	IEPLGVAPTKAK	HexNAc.Hex.NeuAc	100
	724.2	3	2169.6	IEPLGVAPTKAK	HexNAc.Hex.NeuAc_2_	60
JRCSF gp120	512.7	2	1023.4	IEPLGVAPTK	Native peptide	2000
	695.3	2	1388.6	IEPLGVAPTK	HexNAc.Hex	1540
	840.7	2	1679.4	IEPLGVAPTK	HexNAc.Hex.NeuAc	700
	986.4	2	1970.8	IEPLGVAPTK	HexNAc.Hex.NeuAc_2_	1360
	1023.4	2	2044.8	IEPLGVAPTK	HexNAc_2_.Hex_2_.NeuAc	230
	612.6	2	1223.2	IEPLGVAPTKAK	Native peptide	Not Observed
	940.2	2	1878.4	IEPLGVAPTKAK	HexNAc.Hex.NeuAc	340
	724.2	3	2169.6	IEPLGVAPTKAK	HexNAc.Hex.NeuAc_2_	2600
NL4-3 gp120	512.7	2	1023.4	IEPLGVAPTK	Native peptide	3840
	695.2	2	1388.4	IEPLGVAPTK	HexNAc.Hex	6000
	840.6	2	1679.2	IEPLGVAPTK	HexNAc.Hex.NeuAc	4680
	986.1	2	1970.2	IEPLGVAPTK	HexNAc.Hex.NeuAc_2_	180
	1023.1	2	2044.2	IEPLGVAPTK	HexNAc_2_.Hex_2_.NeuAc	400
	612.6	2	1223.2	IEPLGVAPTKAK	Native peptide	300
	940.2	2	1878.4	IEPLGVAPTKAK	HexNAc.Hex.NeuAc	250
	724.2	3	2169.6	IEPLGVAPTKAK	HexNAc.Hex.NeuAc_2_	4100
NL4-3 gp120+gp140	512.7	2	1023.4	IEPLGVAPTK	Native peptide	3900
	695.2	2	1388.4	IEPLGVAPTK	HexNAc.Hex	1490
	840.9	2	1679.8	IEPLGVAPTK	HexNAc.Hex.NeuAc	250
	612.6	2	1223.2	IEPLGVAPTKAK	Native peptide	370
	940.2	2	1878.4	IEPLGVAPTKAK	HexNAc.Hex.NeuAc	100
	724.2	3	2169.6	IEPLGVAPTKAK	HexNAc.Hex.NeuAc_2_	Not Observed

Electrospray Nano-LC-MS/MS summary showing the O-glycan occupancy of the conserved threonine of secreted HIV-1 YU2 gp120, HIV-1 JRCSF gp120, HIV-1 NL4-3 gp120 + gp140 mixture, HIV-1 YU2 gp120 + gp140. m/z is mass to charge ratio. MW is molecular weight. HexNAc.Hex is core 1. HexNAc.Hex.NeuAc is monosialylated core1. HexNAc.Hex.NeuAc2 is disialylated core 1. HexNAc2.Hex.2NeuAc is core 2.

### O-glycosylation of a highly conserved Thr for secreted HA1 of the influenza A virus

**Fig 4:**
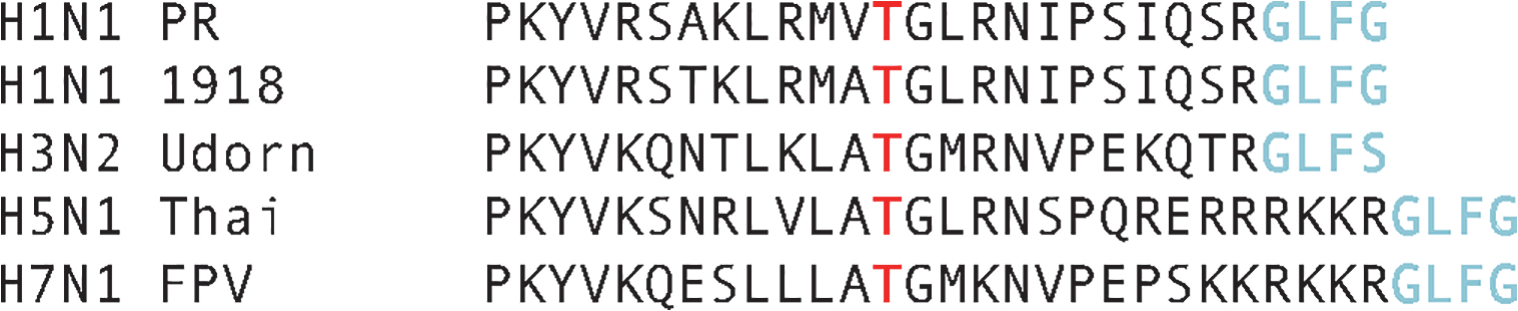
Conservation of a C-terminal threonine in HA1 of influenza A. (A) Alignment of the C-terminus of HA1 and the N-terminus of HA2 for subtypes of influenza A virus. The highly conserved Thr is shown in red. Black letters are amino acids at the C-terminus of HA1. Light blue letters are amino acids at the N-terminus of HA2.

**Fig 5:**
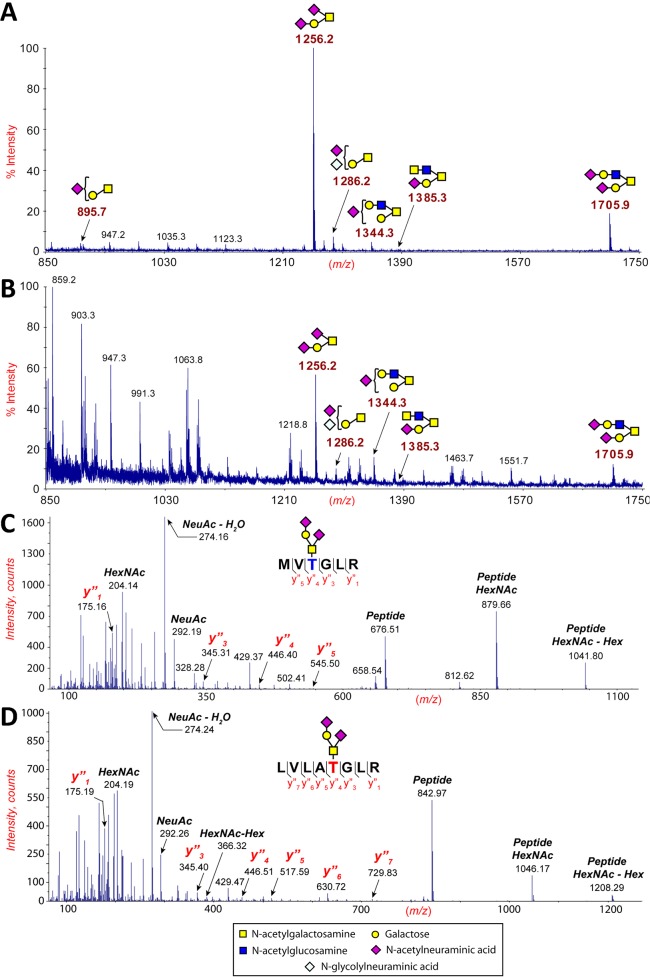
O-glycosylation status of the similarly positioned conserved Thr of influenza A virus H1N1 Puerto RIco HA1 and of influenza A virus H5N1 Thai HA1. (A) MALDI-TOF MS of permethylated O-glycans isolated from recombinant HA1 of Influenza A virus H1N1 Puerto Rico (B) MALDI-TOF MS of permethylated O-glycans isolated from recombinant HA1 of Influenza A virus H5N1 Thai (C) Electrospray Nano-LC-MS/MS spectra showing the location and attachment of the major species of O-glycan attached to Thr318 in HA1 of Influenza A virus H1N1 strain Puerto Rico; parent ion [M+2H]^2+^ m/z 895.48. (D) Electrospray Nano-LC-MS/MS spectra showing the location and attachment of the major species of O-glycan attached to Thr318 in HA1 of Influenza A virus H5N1 strain Thai; parent ion [M+2H]^2+^ m/z 895.48.

Our in silico analyses revealed a similarly-positioned, conserved Thr in HA1 among influenza A viruses (Fig 4). For influenza A virus H1N1 Puerto Rico the corresponding Thr is 12 amino acids from the predicted C terminus of HA1 and for influenza A virus H5N1 Thai the corresponding Thr is 16 amino acids from the predicted C-terminus of HA1. Using the NetOGlyc3.1 algorithm to predict potentially O-glycosylated residues, this conserved Thr of influenza A virus H1N1 Puerto Rico had a G-score of 0.267 and an I-score of 0.056 while the conserved Thr of influenza A virus H5N1 had a G-score of 0.235 and an I-score of 0.123 in the NetOGlyc3.1 algorithm. These scores are not predictive for the attachment of O-linked carbohydrate; however, our glycomic and glycoproteomic analyses of the secreted form of HA1 for both strains of influenza A virus demonstrated that recombinant HA1 is modified with various forms of core 1 and core 2 O-linked carbohydrate (Fig 5A and 5B). In both cases, the secreted HA1 was modified at the conserved Thr 12 amino acids from the predicted C-terminus for influenza A virus H1N1 (Fig 5C) and 16 amino acids from the predicted C-terminus for influenza A virus H5N1 (Fig 5D).

### O-glycosylation status of the highly conserved Thr for secreted HIV-1 gp120+140

We next examined the O-linked carbohydrate content when gp120 was produced by furin cleavage from secreted gp140 (gp120 + ectodomain of gp41). In these analyses, there was no direct way to determine if the O-glycosylated peptide was from gp120 or gp140 since the sample contained a mixture of the gp120 + gp140 proteins. For both HIV-1 NL4-3 and HIV-1 YU2, O-linked carbohydrate was detected (Table 1). However, in contrast to gp120 expressed on its own in the absence of gp41 sequences, where the conserved Thr was almost fully glycosylated, less than 50 percent of the gp120/gp140 mixtures were O-glycosylated (assuming equivalent ionization efficiencies) and in both cases the O-linked carbohydrate chains were mainly the shorter non-and monosialylated core 1 glycans (Table 1).

### Lack of O-glycosylation of the highly conserved Thr on virion gp120 for HIV-1 Bal and SIV CP-MAC

To determine if the highly conserved Thr near the C-terminus of gp120 was modified with O-glycan on virions, HIV-1-BAL and SIV CP-MAC were produced from infected human T-cell lines, virions were purified from culture supernatants, gp120s were HPLC purified with or without additional SDS PAGE purification, and O-linked carbohydrate was analyzed by our glycoproteomic methods. We purified: ~150 μg HIV-1 BAL/SupT1-R5 gp120 by SDS-PAGE and ~ 110 μg by HPLC and SDS-PAGE from 5.2L of cell culture supernatant; we purified ~ 90 μg SIV-CP-MAC/SupT1 gp120 by HPLC from 1.5L of cell culture supernatant. To evaluate the potential impact of HPLC purification on the ability to detect O-glycosylation on Thr residues, we also purified ~ 70 μg recombinant HIV-1 JRCSF gp120 by HPLC. For the virion-derived gp120, the peptides spanning the conserved Thr gave strong molecular ions but no O-glycosylation was observed (Table 2). We are confident that our failure to observe O-glycosylation of the conserved Thr was not due to the purification methods as recombinant HIV-1 JRCSF gp120 purified by the same methods contained the characteristic O-glycosylated Thr residue observed for recombinant proteins. Our failure to observe the O-glycosylation of the C-terminal Thr of gp120 purified from virions was also not due to sensitivity issues or the purification methods because the SIV virion sample yielded high quality data for the O-linked glycopeptides derived from the V1 mucin domain of SIV gp120 [19] and both virion samples yielded a plethora of N-linked glycopeptides.

**Table 2:** HIV-1 Bal and SIV CP-MAC gp120 purified from virions lack O-linked carbohydrate.

Sample	m/z	Charge	Calculated MW	Peptide	Carbohydrate	Approx. Intensity
**HIV-1 BAL gp120**	512.9	2	1023.8	IEPLGVAPTK	Native Peptide	3500
**Purified from virions**	695.3	2	1388.6	IEPLGVAPTK	HexNAc.Hex	Not Observed
	840.7	2	1679.4	IEPLGVAPTK	HexNAc.Hex.NeuAc	Not Observed
	986.4	2	1970.8	IEPLGVAPTK	HexNAc.Hex.NeuAc_2_	Not Observed
** **	1023.4	2	2044.8	IEPLGVAPTK	HexNAc_2_.Hex_2_.NeuAc	Not Observed
**SIV CP-MAC gp120**	783.5	2	1565.0	LVEITPIGLAPTDVK	Native peptide	3100
**Purified from virions**	885.0	2	1768.0	LVEITPIGLAPTDVK	HexNAc	Not Observed
	966.0	2	1930.0	LVEITPIGLAPTDVK	HexNAc.Hex	Not Observed
	1148.6	2	2295.1	LVEITPIGLAPTDVK	HexNAc_2_.Hex_2_	Not Observed
	1294.1	2	2586.2	LVEITPIGLAPTDVK	HexNAc_2_.Hex_2_.NeuAc	Not Observed
	1257.2	2	2512.4	LVEITPIGLAPTDVK	HexNAc.Hex.NeuAc_2_	Not Observed
**HIV NL 4–3 T449S gp120**	505.9	2	1009.8	IEPLGVAP**S**K	Native Peptide	4500
**T>S Mutant**	688.4	2	1374.8	IEPLGVAP**S**K	HexNAc.Hex	Not Observed
	834.0	2	1665.9	IEPLGVAP**S**K	HexNAc.Hex.NeuAc	Not Observed
	979.5	2	1957.0	IEPLGVAP**S**K	HexNAc.Hex.NeuAc_2_	Not Observed
	1016.5	2	2031.0	IEPLGVAP**S**K	HexNAc_2_.Hex_2_.NeuAc	Not Observed

Electrospray Nano-LC-MS/MS summary showing the lack of O-glycan occupancy of the conserved Thr of HIV-1 Bal gp120 and SIV CP-MAC gp120 when protein was purified from virions. Also shown is the lack of O-glycan occupancy on recombinant secreted gp120 of the T499S mutant of HIV-1 strain NL4-3. m/z is mass to charge ratio. MW is molecular weight. HexNAc.Hex is core 1. HexNAc.Hex.NeuAc is monosialylated core1. HexNAc.Hex.NeuAc_2_ is disialylated core 1. HexNAc_2_.Hex_2_.NeuAc is core 2.

## Discussion

We have unambiguously documented glycosylation of the C-terminal threonine of the surface glycoprotein of 5 of 5 strains of HIV-1, 1 of 1 strain of SIV, and 2 of 2 strains of Influenza virus when the protein was made in individual secreted form. Furthermore, we have documented in exquisite detail the nature of the O-linked carbohydrate on these proteins. Others have also recently noted the presence of O-linked carbohydrate on this threonine of HIV-1 gp120 [16,17]. Disialylated core 1 carbohydrate was the predominant form on all eight surface glycoproteins that we examined following purification of a secreted form of each protein.

In stark contrast to the results of our chemical analyses of gp120s made as directly secreted recombinant product from HEK293T cells, purified virions of HIV-1 and SIVmac from productively infected T cell lines were devoid of this O-glycosylation on gp120. It is unlikely that the absence of C-terminal threonine O-glycosylation on gp120 of purified virions is a result of some sort of technical oversight. When secreted gp120 was taken through the same purification steps in parallel with the virion gp120 purification in our experiments, O-glycosylation of the C-terminal threonine was still present. Also, O-linked glycopeptides from the SIV gp120 V1 domain [19] were readily detected on the SIV virion gp120, as were an abundance of N-linked glycopeptides. The lack of O-linked carbohydrate on the conserved Thr on virions is consistent with our finding that secreted gp140 is modified to a lesser extent and with shorter chains of O-glycans than secreted gp120. The results suggest the possibility that the gp41 transmembrane subunit may limit the accessibility of the transferase enzymes that initiate and extend mucin type O-glycosylation. The lack of O-glycosylation of this threonine on HIV and SIV virions is also consistent with the inability to find O-glycosylation of the corresponding threonine in crystal structures of HA1/HA2 purified from influenza virions (Ian Wilson, personal communication). Our results with HIV-1 appear to be at odds with the findings of Yang et al who reported low-level detection of Gal1GalNAc1 on residue 499 of HIV-1 virions [30].

N-linked glycosylation initiates in the ER with the formation of oligomannose glycans. Further glycan processing occurs in the form of mannose trimming, first by mannosidase I, and the formation of complex N-linked carbohydrate as the glycoprotein traverses to and through the golgi. Addition of mucin-type O-linked carbohydrate is a late event occurring in the golgi. It has been suggested that the removal of the outermost mannose residues by mannosidase I from the mannose-rich N-linked glycans is required before O-glycosylation can ensue [31]. The timing of furin cleavage may also be a critical factor in creating a structure that can allow or obviate specific glycosylation events. With regard to HIV-1 gp120, the Scanlan laboratory has noted that HIV-1 virion gp120 is surprisingly dominated by unprocessed oligomannose N-linked glycans, in contrast to gp120 made as the truncated secreted product which is dominated by the more fully processed complex glycans [32,33]. Thus, the nature of N-linked glycosylation events that can be achieved on gp120 appears to depend on whether the gp120 is made directly as a truncated secreted product vs from the gp160 precursor in the formation of virions. It seems likely that our findings are related to these observations from the Scanlan laboratory. Our findings also appear analogous to those of Ahmed et al who noted O-linked glycosylation on proteins expressed as Fc fragments but not when expressed as the authentic IgG [34].
